# Addressing social norms for adolescent timing and spacing of pregnancy in low and middle-income countries: Developing a global research agenda

**DOI:** 10.7189/jogh.14.04206

**Published:** 2024-11-15

**Authors:** Jasmine Uysal, Anvita Dixit, Catherine Green, Marilyn Akinola, Bryan Shaw, Rebecka Lundgren

**Affiliations:** 1 Center on Gender Equity and Health, University of California, San Diego, California, USA; 2 University of Ottawa, Ottawa, Ontario, Canada; 3 Center of Excellence for Infant and Early Childhood Mental Health Consultation, Georgetown University, Washington DC, USA; 4 Center for Global Health Practice and Impact, Georgetown University, Washington DC, USA

## Abstract

**Background:**

Social norms shape adolescent sexual and reproductive health behaviours contributing to contraceptive and pregnancy outcomes. No global research agendas exist to guide research on adolescent social norms shifting in low- and middle-income countries (LMICs). We developed a social norms research agenda to improve adolescent healthy timing and spacing of pregnancy in LMICs.

**Methods:**

We adapted and applied the Child Health and Nutrition Research Initiative (CHNRI) method. A group of researchers guided the process, and consulted with diverse experts to develop a list of 21 research questions for global stakeholders to score via an online survey. Survey participants scored each research question according to four criteria (fills key gap, feasible, impactful, equitable). Research priority scores (RPS) and average expert agreement (AEA) statistics were calculated for each question and analysed overall and by stakeholder region and profession.

**Results:**

We received 185 survey responses. Participants were, on average, 44 years old, 64% were women, 70% were from LMICs and 47% were implementers. The RPS ranged from 52 to 81% (74% median) and the AEA ranged from 49 to 70% (58% median). Nearly 70% of stakeholders gave the same score to each of the top five research questions. The top five research priorities focused on effective norm-shifting interventions (NSIs) strategies, processes and indicators to NSIs, and NSI adaptation and scale-up.

**Conclusions:**

Using a collaborative and rigorous process with diverse representation from LMICs and implementers, we reached consensus on five priority research questions to guide future adolescent social norms research to improve healthy timing and spacing of pregnancy in LMICs.

Adolescent women 15–19 years old have the highest rates of unmet need for contraceptives of any age group (52%) [1,2], increasing their risk of unintended pregnancy [3]. Over 21 million adolescent women in low- and middle-income countries (LMICs) become pregnant each year, most of which are unintended. The majority of these unplanned pregnancies end in abortions which are often unsafe [2]. Complications from pregnancy and birth are one of the leading causes of death among 15–19-year-old females in LMICs [4]; those who do live, on average, have worse social, health, educational, and economic outcomes over the life course [5], furthering global gender inequities [6]. The World Health Organization (WHO) has called for novel and unique strategies specific to adolescents to improve their use of contraceptive methods and prevent adolescent pregnancy as ‘foundational’ to achieving the Sustainable Development Goals (SDGs) [7].

Social norms shape sexual and reproductive health (SRH) behaviours globally, including contributing to the unmet need for contraception and unintended pregnancy among adolescents in LMICs [8–13]. Social norms describe expectations of acceptable behaviour within a community that are enforced by influential individuals or networks (i.e. reference groups). Gender norms are a type of social norms that dictate acceptable behaviours of men and women [14] and have a profound impact on reproductive health. All SRH outcomes are both positively and negatively influenced by social norms, affecting contraceptive and pregnancy decision-making, health service seeking, and sexual activity [12]. Documented examples of norms in LMICs that contribute to adolescent unmet need for contraceptives include norms that discourage sex and contraceptive use before marriage and promote girl child marriage and early child bearing [15–17]. Norms that are associated with increased adolescent contraceptive use include positive attitudes towards contraceptives and/or adolescent networks where contraceptive use is common [9,18]. Importantly, adolescents who do not comply with prevailing norms may face backlash [19].

Evidence is building on the power of norm-shifting interventions (NSIs), an emerging focus within social and behaviour change (SBC) programmes focused on healthy timing and spacing of pregnancy, to improve contraceptive outcomes [12]. These initiatives engage with the community and social systems to uplift social norms that support behaviours which lead to positive health. Several NSIs in sub-Saharan Africa using media and/or couple and community dialogues have shown positive effects on increasing contraceptive use [12]. Two of these interventions, one in rural Niger including in-person home visits with couple counselling and same sex group discussions and another in Palestine using a mobile text messaging intervention, focused directly on adolescents and young women, both finding evidence of increased norms and behaviour supporting contraceptive use [18,20-23]. While these interventions show promise, more evidence is needed to understand which social norms most directly affect adolescent behaviour, what strategies, and combinations of strategies, are most effective in shifting norms to improve both contraceptive and pregnancy outcomes, and how to sustain and scale this change for adolescents in LMICs [12].

Research agendas have been utilised across disciplines to identify research priorities for a particular population and outline a clear framework for making strategic decisions. Recently, a research agenda was developed by the Research for Scalable Solutions (R4S) project on family planning across six countries that highlights the importance of understanding and intervening on norms for contraceptive ‘acceptability and uptake’ [24]. No global stakeholder agendas have yet been developed, however, which focus on adolescent and youth social norms research.

This paper describes the process and results of developing a global public health social norms research agenda to improve adolescent healthy timing and spacing of pregnancy in LMICs. This initiative constitutes the concluding deliverable of The Passages Project, an implementation research project spanning from 2015–2022 which aimed to address a broad range of social norms, particularly among adolescents and youth, to sustainably improve reproductive health and gender equality at scale. The research agenda aims to reach a broad range of stakeholders, including funders, researchers, implementers, and policy makers, to crystalise and catalyse the next phase of social norms implementation research to achieve improvements in youth SRH by 2030.

## METHODS

**Figure 1:**
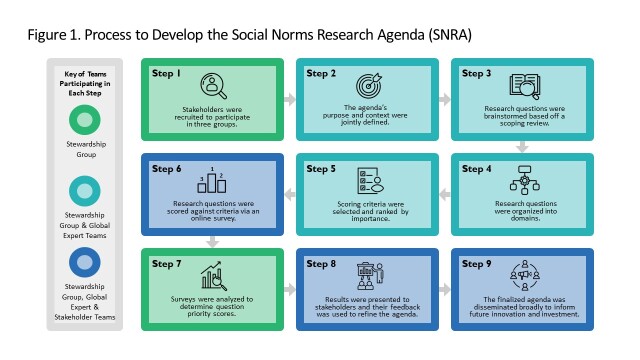
Process to develop the social norms research agenda.

To develop this research agenda, we adapted and applied the Child Health and Nutrition Research Initiative (CHNRI) method [25]. The CHNRI method was first developed and used in 2007 to create research priorities for child health and has since been adapted and applied to over 50 research agendas globally [26]. This method empirically crowdsources global stakeholder opinions through online surveys to systematically set research agenda priorities based on stakeholders’ ratings [27]. For this research agenda, we adapted and applied CHNRI – our methodology is described below and visualised in **Figure 1**.

### Identification and recruitment of stakeholders and experts to participate

Individuals were recruited to participate in the development of this research agenda in three groups – the Stewardship, Expert, and Stakeholder groups (**Figure 1**).

The Stewardship Group, comprised of authored researchers, was involved in all steps and guided the overall process of developing the agenda. Specific tasks unique to this group included designing the research methodology, recruiting stakeholders in each group, facilitating meetings, developing and disseminating the surveys, and analysing research question priority scores (**Figure 1**). The group and project was led by a implementation science researcher with over 30 years of experience and prior expertise in identifying best practices and setting research priorities for global public health and development and social norms programmes.

The Expert Group consisted of 34 expert volunteers working on norm-shifting related to adolescent and youth healthy timing and spacing of pregnancy. They provided input into defining the context of the research agenda and the design and development of the research question priority scoring survey (**Figure 1**) including determining research questions to be scored, the criteria research questions were to be scored against, and survey testing. To recruit experts, the Stewardship Group developed a list of 44 multi-sectoral professionals across high income countries (HICs) and LMICs based on prior adolescent and youth social norms shifting initiatives, many of whom were involved in the Passages project. Approximately 77% of invited professionals accepted the invitation to join the Expert Group; 15 participants (44.1%) were from LMICs and 19 (55.9%) from HICs. The group was composed of 10 researchers, 18 implementers, four donors, and two policymakers across 28 organisations.

The Stakeholder Group comprised 185 individuals working in adolescent and youth sexual and reproductive health who volunteered to participate in the research question priority scoring survey (**Figure 1**). The survey was distributed from the Stewardship Group to the Expert Group via email. The Stewardship Group forwarded the invitation to personal contacts and also asked individuals in the Expert Group to forward the survey to their personal contacts in a snowball sampling approach. The survey was also distributed to participants through listservs focusing on social norms research and programming and adolescent and youth SRH (e.g. Passages newsletter, Interagency Gender Working Group newsletter). Finally, the survey was posted to social media accounts for the Passages project including Twitter and LinkedIn. The Stakeholder group was also invited, via the same recruitment channels, to participate in a presentation on the draft agenda were the project team elicited feedback and distributed the final research agenda for dissemination (**Figure 1**).

### Identification of research questions and development of stakeholder survey

Research questions for prioritisation were developed and proposed by the Stewardship group in consultation with the Expert Group based on the results of an unpublished scoping review, conducted as part of a separate activity under the Passages project, to examine the existing evidence for norms-shifting healthy timing and spacing of pregnancy research among adolescents (aged 10–19) and young adults (aged 20–35) in LMICs (**Figure 1**). The review searched four databases including PubMed, Embase, CINAHL, and PsychINFO for any articles published from January 2010 through June 2021 in LMICs using search terms focused on family planning and contraception, violence against women and children, child marriage and pregnancy, girls’ education and gender roles, sex education and menstrual health, and hygiene. Articles were uploaded to the literature review software, Covidence [28]. Over 11 550 articles were identified through this initial round of review, of which 7127 non-duplicates were retained (duplicates were removed automatically and manually). Exactly 6697 articles were excluded during title and abstract screening, and 430 articles were retrieved for full text screening; 290 articles were removed for non-relevance during full-text screening, leaving a total of 140 relevant articles. The remaining articles were extracted and summarised in an Excel table by a team of researcher staff and validated by a project scientist with experience conducting systematic literature reviews. Research questions for articles having anything to do with healthy timing and spacing of pregnancy, primarily those focused on family planning and contraception, were then selected to inform the social norms research agenda.

The review identified 101 articles related to this activity whose research questions were then categorised into four domains based on Passages project areas of interest (**Figure 1**). These domains include:

1) understanding social norms

2) monitoring and evaluation of NSIs

3) design and implementation of NSIs

4) adaptation and scale-up of NSIs.

Approximately 83% of articles included research questions related to domain 1, 39% of articles included research questions related to domain 2, 15% of articles included research questions related to domain 3, and, 10% of articles included research questions related to domain 4. Overall, the vast majority of the articles identified in the scoping review were exploratory, observational, or descriptive rather than evaluative. There were few examples of effective NSIs for healthy timing and spacing of pregnancy among adolescents.

Review findings were presented to the Expert Group in a webinar where experts broke into small groups and brainstormed research questions to fill identified research gaps within each domain. The Stewardship Group reviewed the listing from the webinar, clarified and consolidated similar questions, and distributed the listing of research questions to the Expert Group via email, allowing additional questions to be proposed over a two-week commenting period. Originally, 35 questions were proposed, which were consolidated into 21 questions included in the final survey.

On a subsequent webinar, the expert group also determined the four criteria that research questions would be scored against on the survey. A listing of the original CHNRI scoring criteria, with small edits for relevance, were presented to the Expert group. The Expert group discussed the number of criteria to be adopted (four) and used menti-meter to vote on which criteria were most important to include. Finally, the group discussed and finalised the phrasing of the scoring criteria (**Figure 1**)

The stakeholder survey was constructed in Qualtrics and distributed to selected members from the Expert Group for pretesting prior to translation to French and Spanish and distribution to the broader Stakeholder Group for research question priority scoring.

### Prioritisation of research questions

The Stakeholder Group was asked to score the refined list of research questions via an online survey (**Figure 1**). The survey, administered in English, French, and Spanish, asked participants to provide general non-identifying demographics, rank the scoring criteria in order of importance, score each of the 21 research questions, and suggest other questions (if needed). Participants could only click on the survey link and complete the survey one time from any single device. Five additional research questions were proposed, and upon further review, were found to overlap with existing questions. Each of the 21 final questions was scored against four criteria, also determined by the Expert Group.

1) This research question fills a key gap.

2) This research question is feasible to answer.

3) This research question is likely to have a high impact.

4) This research question is likely to increase equity.

Scores for each criterion were on a 3-point response scale: ‘yes’ (1), ‘no’ (0), and ‘possibly’ (0.5). The survey was disseminated through Expert Group members, listservs, and social media in June 2022, targeting LMIC and implementing stakeholders. Survey responses were analysed by researchers in the Stewardship Group to create priority scores (**Figure 1**). Priority scores were used to create a draft research agenda focusing on questions with the highest priority scores across and within each of the four domains. The draft research agenda was presented back to all stakeholders who participated in the survey via an online webinar to provide feedback (**Figure 1**) which was used to refine the final product.

### Data analysis

To determine priority questions, we followed the data analysis methodology as guided by Rudan et al. [25]. First, we calculated descriptive statistics to summarise the sample of survey participants. Then, we calculated an intermediate score representing the range of how much the proposed research questions satisfy the priority-setting criteria. We added all the non-blank responses (i.e. ‘1’, ‘0.5′, or ‘0’) across the four ranking criteria and then divided this sum by the number of received answers. Thus, a value of 0 to 100% was assigned for all intermediate scores. Missing responses were left out of the numerator and denominator.

Next, Research Priority Scores (RPS) were calculated for each question. As described above, stakeholders were also asked to rank the four scoring criteria based on importance on a scale of one (most important) to four (least important) to develop weights for the calculation of the RPS. To aid in interpretation, rankings were then inverted so that higher scores represented more important criteria. The intermediate scores for each criterion were multiplied by weights developed from stakeholder ranking of scoring criteria summed across all four scoring criteria and divided by the sum total rank order of the scoring criteria. The research questions were then ordered by priority according to their RPS. The following formula was used to calculate the RPS:



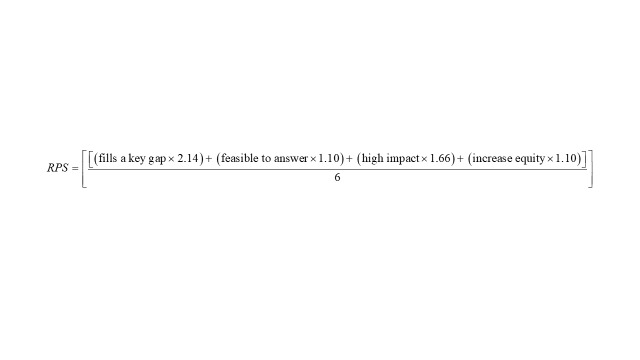



We then calculated the Average Expert Agreement (AEA) scores to represent the average proportion of scorers that agreed on the four criteria questions asked for each research question [29]. For each evaluated research question the AEA informs, for an average question, what proportion of scorers gave the same most frequent answer. The following formula was used to calculate the AEA:



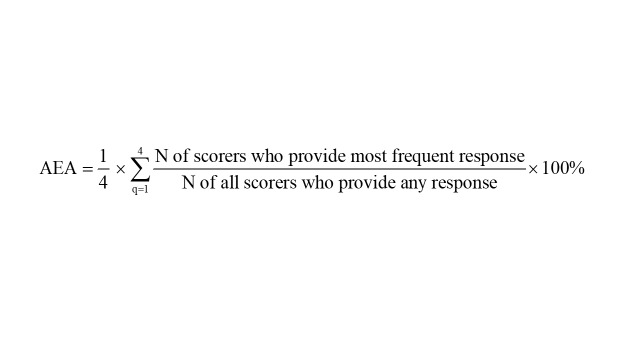



Analyses were stratified by region to determine differences in scores between respondents from different stakeholder global regions (sub-Saharan Africa, North America, South Asia, Latin America, Europe, East Asia/Pacific, Eastern Europe/Central Asia), and different stakeholder professionals (researchers/academics, programme implementers, donors/policymakers, and undisclosed). All data analysis was performed using Microsoft Excel, 2018 (https://office.microsoft.com/excel).

### Dissemination

The final agenda, after refinement based on stakeholder feedback, was disseminated using a social media campaign, the same listservs used to recruit participants, and through personnel networks of the Stewardship and Expert Groups. The final agenda is now available for the public on USAID’s (https://pdf.usaid.gov/pdf_docs/PA00ZKW4.pdf) and the Institute for Reproductive Health’s (https://www.irh.org/resource-library/social-norms-research-agenda/) websites.

### Evaluation of the research priority setting process

Evaluation of the process we used to set these research priorities was integrated into the existing data collection efforts. First, we asked the Stakeholder group participating in the survey to provide feedback into the process at the end of the survey. We asked individuals if they had comments about the research questions proposed, or any other feedback about the research questions, criteria, agenda, or the survey itself. We also gathered feedback from stakeholders who participated in the final webinar via group discussion and menti-meter word clouds and open response feedback.

### Research ethics

This study was exempted from human subjects review by the University of California, San Diego (UC San Diego) Institutional Review Board under 45 CFR.104(d) category 2. Identifying information (beyond gender, region, professional type and organisational type) was not collected from stakeholders participating in the online survey.

## RESULTS

### Sample description

We received 288 ‘clicks’ on the survey, and 185 responses where at least one research question was scored. A majority of the surveys, 135 (73%), were completed in English, 42 (23%) in French and 8 (4%) in Spanish. Stakeholders who took the survey were, on average, 44 years old (range 22 to 73 years). A majority of stakeholders (64%) identified as women, 33% identified as men, and only 3% identified as another gender identity. Seven out of 10 stakeholders (n = 129) were from LMICs while 30% (n = 52) were from HICs (with two undisclosed). In terms of their profession, most stakeholders were implementers (47%), followed by researchers/academics (39%), donors/policymakers (6%), and undisclosed (8%). Stakeholders participated from seven global regions. This included 51% from sub-Saharan Africa (30% Francophone Africa, 21% Anglophone Africa), 30% from North and South America/Caribbean, 5% from Europe/Central Asia, 14% from South and East Asia/Pacific.

### Priority ranking of criteria

We asked stakeholders to rank-order criteria based on importance, with a rank of four being the highest importance and one the lowest importance. ‘This research question fills a key gap’ was ranked the most important (average rank = 2.14), followed by ‘is likely to have a high impact’ (average rank = 1.66). Criteria on ‘is feasible to answer’ (avg. rank 1.10) and ‘is likely to increase equity’ (average rank = 1.10) were ranked slightly lower.

### Priority research question scores resulting from analysis of online surveys

The RPS of the 21 research questions ranged from 52 to 81% with a median score of 74% (Table S1 in the **Online Supplementary Document**). The AEA ranged from 49 to 70% with a median score of 58%. For the top five research questions, the AEA was between 62 to 70%. Overall, almost seven out of ten stakeholders gave the same score to each of the research questions for the top five priorities. The five top scoring research questions across domains were from domains (2) designing and implementing NSIs, (3) enhancing NSI monitoring and evaluation, and (4) scaling and sustaining NSIs (**Table 1**). We also identified the top three scores questions within each domain (**Table 2**). 

**Table 1:** Research questions with the five highest rankings across domains

Ranking*	Domain	Research question	Overall RPS†	AEA‡
1	2: Designing & implementing NSIs	What interventions (strategies, activities) are effective in shifting norms related to adolescent and youth sexual and reproductive health behaviors/outcomes in LMICs?	81%	70%
2	2: Designing & implementing NSIs	What are effective strategies to engage reference groups (i.e. gatekeepers) to support shifts in norms related to adolescent and youth sexual and reproductive health in LMICs?	80%	67%
3	3: Enhancing NSI monitoring and evaluation	What are practical and valid indicators and approaches to monitor norms-shifting related to adolescent and youth sexual and reproductive health interventions in LMICs?	80%	68%
4	4: Scaling and sustaining NSIs	What are best practices for adapting norms-shifting interventions for adolescent and youth sexual and reproductive health in LMICs?	79%	65%
5	4: Scaling and sustaining NSIs	What are different approaches for scaling sexual and reproductive health norms- shifting interventions for adolescents and youth across sectors (e.g. health, education, community) in LMICs?	78%	62%

AEA – Average Expert Agreement, LMIC – low- and middle-income country, NSI – norm-shifting intervention, RPS – Research Priority Scores

*Research questions ranked according to the RPS across all domains.

†RPS was calculated by multiplying average stakeholder intermediate scores for each question on each of the four criteria and the average stakeholder rank of criteria in order of importance (4 being most important, 1 being least important), divided by the total sum of weighted ranks. Those questions with the same RPS were taken out two decimal places to determine what would be higher ranked.

‡AEA was calculated by multiplying ¼ by the sum of all four criteria of the number of scores who provided the most frequent weighting response divided by the number of scorers who provided any response and multiplied by 100%.

**Table 2:** Research questions with the three highest rankings within each domain

Rank*	Research question	Overall RPS†	AEA‡
	**Domain 1: understanding norms and their influence**		
1	How do norms influence sexual and reproductive health behavior/outcomes differently depending on people's intersecting identities (e.g. socio-economic status, religion, disability, etc.) for adolescents and youth in LMICs?	76%	61%
2	How do social norms influence adolescent and youth sexual and reproductive health behaviors and outcomes in LMICs?	74%	59%
3	How do social norms related to adolescent and youth sexual and reproductive health shift at critical life course transitions (e.g. puberty, sexual debut, marriage, becoming a parent) in LMICs?	72%	55%
	**Domain 2: designing & implementing norm-shifting interventions**		
1	What interventions (strategies, activities) are effective in shifting norms related to adolescent and youth sexual and reproductive health behaviors/outcomes in LMICs?	81%	70%
2	What are effective strategies to engage reference groups (i.e. gatekeepers) to support shifts in norms related to adolescent and youth sexual and reproductive health in LMICs?	80%	67%
3	How can adolescent and youth norm shifting initiatives on sexual and reproductive health engage a diverse consortium of stakeholders (e.g. community individuals and orgs partners, private and public organizations, governments) in intervention design and delivery in LMICs?	74%	56%
	**Domain 3: enhancing norms-shifting intervention monitoring and evaluation**		
1	What are practical and valid indicators and approaches to monitor norms-shifting related to adolescent and youth sexual and reproductive health interventions in LMICs?	80%	68%
2	What are practical and rigorous approaches (e.g. social network analysis, influence mapping, vignettes) to evaluate sexual and reproductive health norms-shifting interventions among adolescents and youth in LMICs?	76%	61%
3	How can we assess the costs and cost-effectiveness of norms-shifting interventions to improve adolescents and youth sexual and reproductive health in LMICs?	73%	58%
	**Domain 4: scaling and sustaining norms-shifting interventions**		
1	What are best practices for adapting norms-shifting interventions for adolescent and youth sexual and reproductive health in LMICs?	79%	65%
2	What are different approaches for scaling sexual and reproductive health norms- shifting interventions for adolescents and youth across sectors (e.g. health, education, community) in LMICs?	78%	62%
3	What program characteristics (e.g. frequency, duration, technologies utilized, strategies, participants, facilitators) facilitate scale up of sexual and reproductive health norms-shifting interventions for adolescents and youth in LMICs? How and why do they do so?	76%	60%

AEA – Average Expert Agreement, LMIC – low- and middle-income country, NSI – norm-shifting intervention, RPS – Research Priority Scores

*Research questions ranked according to the RPS within each of the four domains.

†RPS was calculated by multiplying average stakeholder intermediate scores for each question on each of the four criteria and the average stakeholder rank of criteria in order of importance (4 being most important, 1 being least important), divided by the total sum of weighted ranks. Those questions with the same RPS were taken out two decimal places to determine what would be higher ranked.

‡AEA was calculated by multiplying ¼ by the sum of all four criteria of the number of scores who provided the most frequent weighting response divided by the number of scorers who provided any response and multiplied by 100%.

### Differences by stakeholder characteristics

**Figure 2:**
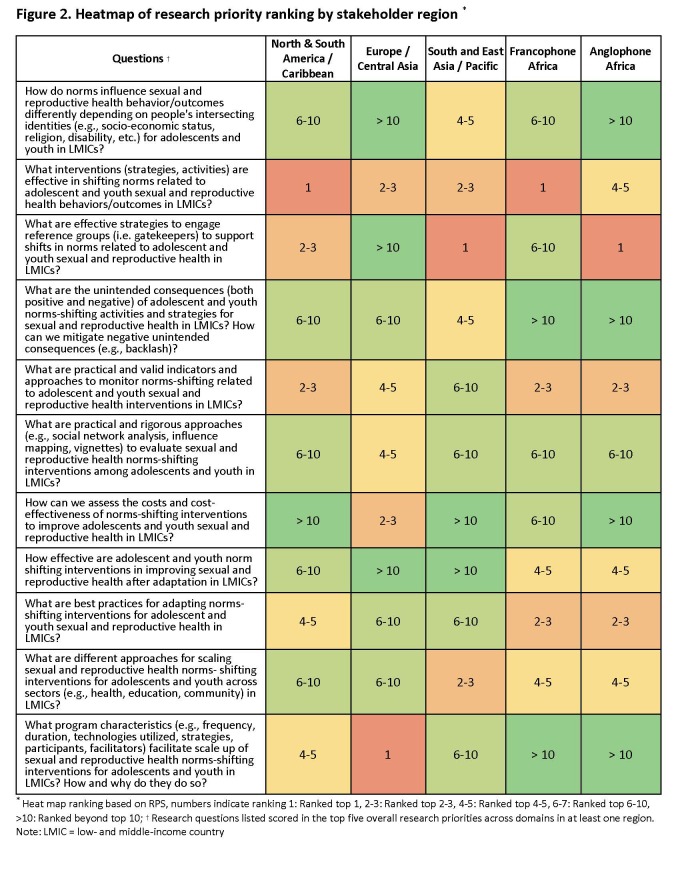
Heatmap of research priority ranking by stakeholder region. *Heat map ranking based on RPS, numbers indicating ranking: 1: Ranked top 1, 2–3: Ranked top 2–3, 4–5: Ranked top 4–5, 6–7: Ranked top 6–10, >10: Ranked beyond top 10. †Research questions listed scored in the top five overall research priorities across domains in at least one region. LMIC – low- and middle-income country, RPS – research priority scores.

**Figure 3:**
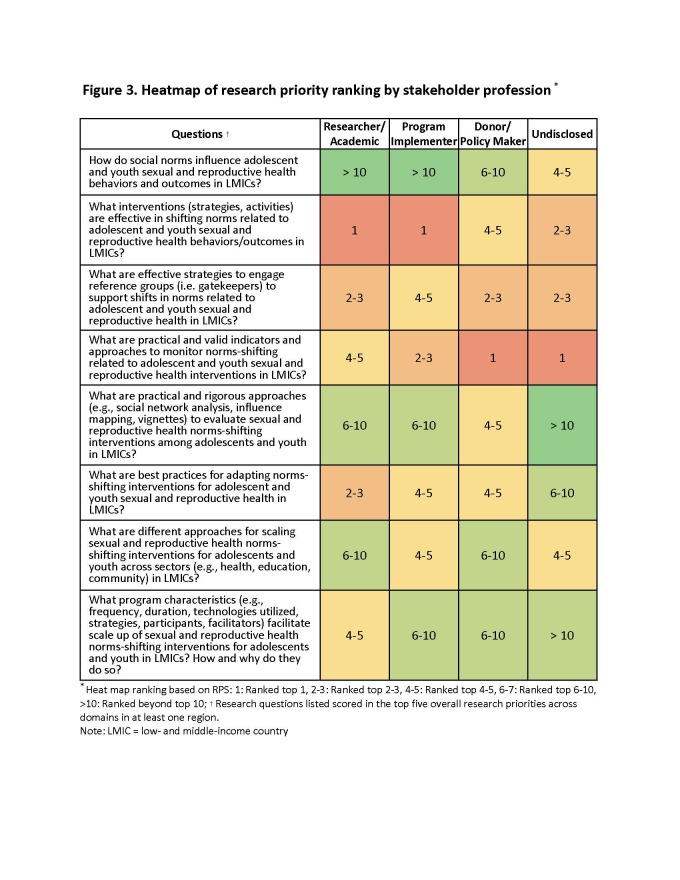
Heatmap of research priority ranking by stakeholder profession. *Heat map ranking based on RPS: 1: Ranked top 1, 2–3: Ranked top 2–3, 4–5: Ranked top 4–5, 6–7: Ranked top 6–10, >10: Ranked beyond top 10. †Research questions listed scored in the top five overall research priorities across domains in at least one region. LMIC – low- and middle-income country, RPS – research priority scores.

We observed variability in scoring across stakeholder region and profession groups, as illustrated in **Figure 2** and **Figure 3**. Across all regions, the research question ‘What interventions (strategies, activities) are effective in shifting norms related to adolescent and youth sexual and reproductive behaviours/outcomes?’ ranks in the top five priorities. In Europe and Central Asia, stakeholders ranked questions on programme characteristics for scale-up and cost/cost-effectiveness as top priorities, while they scored slightly lower in other regions. Sub-Saharan Africa stakeholders consistently ranked questions around adaptation in the top five, while these were ranked slightly lower in other regions. In Asia and Anglophone Africa and North and South America, strategies to engage reference groups were in the top three priorities but ranked lower in Europe/Central Asia and Francophone Africa.

Across all professions, three questions ranked in the top five priorities, including:

1) effective interventions to shift norms

2) effective strategies to engage reference groups to support shifts in norms

3) practical and valid indicators and approaches to monitor norms shifting.

Academics ranked best practices for adaptation among their top three priorities, while other professions ranked this in their top 10. Programme implementers ranked scale up questions in their top five, while academics and donors/policymakers scored them slightly lower.

### Refinements and changes based on stakeholder and expert feedback

In the final webinar and on the research question priority scoring survey, we gathered feedback from participants asking for suggestions to improve the research agenda or process of developing the research agenda. Suggestions received included: focusing on youth participation in research, avoiding specialist terminology, and, in future initiatives, improving the equity scoring criteria which some found confusing. Others found some of the research questions too general and in need of cultural contextualisation. To address these concerns, the Stewardship group added two cross-cutting questions to:

1) better understand cultural considerations

2) better understand approaches and the impact of involving young people in designing and implementing research.

We also further clarified and defined terms to make the document and questions more accessible. Other suggestions are addressed in the limitations.

## DISCUSSION

Through this process, global experts reached a consensus on five high-priority questions in social norms research to advance adolescent and youth healthy timing and spacing of pregnancy. These questions prioritise effectiveness and implementation research to inform programmes and policy, rather than generating exploratory or descriptive knowledge of social norms generally. A large and diverse group of global stakeholders agreed that the priority is to conduct research on ‘what works’ to design, implement, and scale effective NSIs. This priority is consistent with the research questions proposed in the USAID High Impact Practice (HIP) brief on promoting community support for family planning through social norms interventions [12]. Additionally, in a recently developed family planning research agenda across six countries, stakeholders highlighted the importance of understanding norms for adolescents specifically, prioritising two research questions on interventions to improve adolescent contraceptive uptake and involving reference groups that may positively or negatively impact SRH decision-making [24], questions which emerged as the top two research priorities through this initiative.

While the global community is becoming increasingly aware of the importance, power, and promise of social-norms shifting approaches to achieve desired social and behaviour change, researchers and practitioners adopting social norms-shifting approaches must also be aware of the potential pitfalls of this approach, including unintentionally promoting negative social norms [30–32]. Ethical approaches to addressing social norms require sensitivity to power and meaningful community engagement in design and implementation, including monitoring and responding to unintended and intended effects. This requires collaborative efforts based on foundations of transparency and equity which ensure that all voices, including marginalised groups, are engaged [30,32]. Effective monitoring of social norms efforts, with community members and frontline workers as active participants, combined with continuous monitoring, learning, and improvement will allow teams to identify and mitigate unexpected negative consequences and encourage positive changes. Although our research question focused on unintended consequences did not rank in the top five via our CHNRI process, research should prioritise exploring unintended consequences and engaging local voices to ensure results reflects local meaning and perspectives.

There is increasing recognition of the importance of ‘localising’ global goals and priorities to ensure that results are relevant and meaningful for local action [33]. This research agenda, systematically constructed based on a rigorous understanding of the evidence and input from diverse stakeholders, provides one tool to prioritise research initiatives based on evidence gaps and local need, thereby improving the likelihood that research results will be utilised in ways that have an impactful improvement on adolescent health. While these research questions may help guide general goals and priorities for global health research in LMICs, contextualisation based on the research target audience and region will be required in their application. This analysis is one of the few CHNRI processes to present research priorities, similarities and differences by both stakeholder region and profession [34]. While many priorities were similar, important differences emerged that should be considered when designing and funding research to ensure generated knowledge is relevant to decision-makers in target regions/disciplines. For example, in Europe/Central Asia region, the research question ‘What program characteristics (e.g. frequency, duration, technologies utilised, strategies, participants, facilitators) facilitate scale up of sexual and reproductive health norms-shifting interventions for adolescents and youth in LMICs? How and why do they do so?’ scored as the top priority while it ranked as a lower priority in other regions where higher priority questions focused on identifying effective interventions. This difference could suggest that funders, researchers, and technical advisors focused in higher-income regions, like Europe, could have a greater focus on mechanisms of scaling than stakeholders in LMICs who may be in earlier stages of identifying and implementing effective interventions (required prior to scaling). Though we are not aware of others that have yet utilised this information to tailor research agendas, we posit that if focusing on a particular region, contextualising research priorities to that region could produce research that is more likely to be applied to programme and policy initiatives.

When applying the CHNRI method for this activity, the authors simplified the approach to ensure the process was accessible to a diverse group of participating stakeholders while retaining the overall structure. Prior studies applying CHNRI have also adapted the approach to fit within the particular context and subject of interest [25,26]. We found particular aspects of CHNRI, such as selection of scoring criteria, were not intuitive to some implementing and policy stakeholders. Despite several rounds of review and input, several stakeholders found the criteria of equity difficult to apply without considering specific study design. Other approaches exist to set global research priorities [35], which may be more intuitive, but may also require more resources. The CHNRI method, however, has some advantages over discussion-based methods due to its replicability, ability to reach large numbers of stakeholders, weigh stakeholder opinions equally, and systematically assess and contextualise results.

Despite the strengths of our approach, there were limitations to the process. Importantly, our process did not engage youth due to resource and time limitations. Globally, there is an increasing emphasis on directly involving youth in planning and implementing research and programmes to improve adolescent SRH [36–39]. Future work to contextualise and implement this research agenda should seek to directly engage with young people within target communities. Additionally, government professionals were underrepresented in this activity, and targeted efforts are needed to engage them in research agenda setting moving forward. Due to our snowball sampling approach and anonymous survey, it was not possible for us to track the success of our recruitment efforts nor from where the majority of participants stakeholders were recruited. We also did not provide guidance nor evaluate how successfully this research agenda was in moving actual research funding allocation in global development efforts. Finally, Spanish speaking and other gender identity stakeholders were underrepresented. Future research and advocacy efforts should centre their efforts with these groups as priorities are likely to shift based on stakeholders involved.

## CONCLUSIONS

The social norms research agenda described here offers consensus-based global priorities for research investment in social norms research in the area of adolescent and youth healthy timing and spacing of pregnancy. Vetted across regions and disciplines, these research priorities provide a roadmap for researchers who wish to advance knowledge and, subsequently practice, of the design, implementation, monitoring, adaptation and scale of norm-shifting interventions in ways which resonate with global and local stakeholders.

## Additional material


Online Supplementary Document
