# Supplementary material for: Addressing social norms for adolescent timing and spacing of pregnancy in low and middle-income countries: Developing a global research agenda
Source: J Glob Health. 2024 Nov 15;14:04206. doi: 10.7189/jogh.14.04206 (PMC11565465; doi:10.7189/jogh.14.04206)
Supplement: Online Supplementary Document [file jogh-14-04206-s001.pdf]

Supplementary Data

Table S1. Ranking by RPS for all 21 research questions across domains.

Overall rank	Domain	Research question	RPS	AEA
1	2. Designing & implementing NSIs	What interventions (strategies, activities) are effective in shifting norms related to adolescent and youth sexual and reproductive health behaviors/outcomes in LMICs?	81%	70%
2	2. Designing & implementing NSIs	What are effective strategies to engage reference groups (i.e. gatekeepers) to support shifts in norms related to adolescent and youth sexual and reproductive health in LMICs?	80%	67%
3	3. Enhancing NSI monitoring and evaluation	What are practical and valid indicators and approaches to monitor norms-shifting related to adolescent and youth sexual and reproductive health interventions in LMICs?	80%	68%
4	4. Scaling and sustaining NSIs	What are best practices for adapting norms-shifting interventions for adolescent and youth sexual and reproductive health in LMICs?	79%	65%
5	4. Scaling and sustaining NSIs	What are different approaches for scaling sexual and reproductive health norms-shifting interventions for adolescents and youth across sectors (e.g., health, education, community) in LMICs?	78%	62%
6	1. Understanding norms and their influence	How do norms influence behavior/outcomes differently depending on people's intersecting identities (e.g., socio-economic status, religion, disability, etc.) for adolescents and youth in LMICs?	76%	61%
7	3. Enhancing NSI monitoring and evaluation	What are practical and rigorous approaches (e.g., social network analysis, influence mapping, vignettes) to evaluate adolescent and youth norms-shifting interventions in LMICs?	76%	61%
8	4. Scaling and sustaining NSIs	How effective are adolescent and youth norm shifting interventions after adaptation?	76%	60%
9	4. Scaling and sustaining NSIs	What program characteristics (e.g., frequency, duration, technologies utilized, strategies, participants, facilitators) facilitate scale up of sexual and reproductive	76%	60%

		health norms-shifting interventions for adolescents and youth in LMICs? How and why do they do so?		
10	1. Understanding norms and their influence	How do social norms influence adolescent and youth sexual and reproductive health behaviors and outcomes?	74%	59%
11	2. Designing & implementing NSIs	How can adolescent and youth norm shifting initiatives engage a diverse consortium of stakeholders (e.g., community individuals and orgs partners, private and public organizations, governments) in intervention design and delivery?	74%	56%
12	2. Designing & implementing NSIs	What are the unintended consequences (both positive and negative) of adolescent and youth norms-shifting activities and strategies in LMICs? How can we mitigate negative unintended consequences (e.g., backlash)?	73%	57%
13	3. Enhancing NSI monitoring and evaluation	How can we assess the costs and cost-effectiveness of norms-shifting interventions for adolescents and youth in LMICs?	73%	58%
14	1. Understanding norms and their influence	What meta-norms (e.g., authority, gender ideology, control & violence) influence adolescent and youth sexual and reproductive health	72%	56%
15	3. Enhancing NSI monitoring and evaluation	What are reliable and valid measures of social norms constructs (e.g., descriptive norms, injunctive norms, sanctions) for adolescents and youth?	72%	56%
16	4. Scaling and sustaining NSIs	How does scaling adolescent and youth norms-shifting interventions differ with public (e.g., government) vs. private (e.g., non-governmental organization/faith-based organization) sector partners?	71%	52%
17	1. Understanding norms and their influence	How do social norms diffuse through social networks in different contexts?	70%	51%
18	2. Designing & implementing NSIs	How can adolescent and youth sexual and reproductive health norms shifting interventions effectively catalyze diffusion of effects beyond project participants?	69%	56%

19	1. Understanding norms and their influence	Which social norms constructs (e.g., descriptive norms, injunctive norms, sanctions) are related to adolescent and youth sexual and reproductive health behaviors/outcomes?	68%	49%
20	2. Designing & implementing NSIs	Do planned diffusion strategies for adolescent and youth norms shifting interventions result in greater change than spontaneous diffusion?	66%	53%
21	1. Understanding norms and their influence	How do social norms related to adolescent and youth sexual and reproductive health shift at critical life course transitions (e.g., puberty, sexual debut, marriage, becoming a parent)?	52%	55%

RPS: Research Priority Scores, AEA: Average Expert Agreement, AYSRH: Adolescent and Youth Sexual and Reproductive Health, SES: Socio-Economic Status, NGO: Non-Governmental Organization, FBO: Faith-Based Organization
